# Economic Hardship and Health Within Sociodemographic and Occupational Groups — Behavioral Risk Factor Surveillance System, United States, 2022–2023

**DOI:** 10.15585/mmwr.mm7419a3

**Published:** 2025-05-29

**Authors:** Sharon R. Silver, Jia Li, Taylor M. Shockey

**Affiliations:** 1 Division of Field Studies and Engineering, National Institute for Occupational Safety and Health, CDC.

**Summary**

**What is already known about this topic?**

Economic hardships can limit workers’ ability to prevent and address adverse health conditions.

**What is added by this report?**

In this exploratory analysis of 2022–2023 survey data, 6.9% of currently employed or recently unemployed U.S. adults in 36 states and the U.S. Virgin Islands reported at least four of eight economic hardship indicators, suggesting a high level of economic hardship, and 12.5% reported having fair or poor health. Compared with prevalences among all workers combined, prevalences of lacking health insurance and of cost preventing needed medical care were elevated for all but one occupational group with a high level of economic hardship. Workers in occupational groups with a high level of economic hardship were more likely to report fair or poor self-rated health.

**What are the implications for public health practice?**

Policymakers and public health practitioners might develop prevention and intervention strategies tailored to occupational groups with high levels of economic hardships to enhance health.

## Abstract

Economic hardship can limit the ability of workers to prevent and address adverse health conditions. Using 2022 and 2023 Behavioral Risk Factor Surveillance System data, this exploratory analysis assessed economic hardship measures and self-rated health among currently employed and recently unemployed (<12 months) U.S. adults. Measures of economic hardship were 1) employment instability, 2) food insecurity, 3) housing insecurity, 4) utility insecurity, 5) lack of reliable transportation, 6) receipt of food stamps or Supplemental Nutrition Assistance Program benefits, 7) lack of health insurance, and 8) cost as a barrier to needed medical care. Overall, 6.9% of currently or recently employed U.S. adults in 36 states and the U.S. Virgin Islands had high levels of economic hardship (reporting at least four of eight economic hardship indicators), and 12.5% reported having fair or poor health. High levels of economic hardship were more common among persons who were recently unemployed, were aged 18–49 years, were female, were Hispanic or Latino (Hispanic) or non-Hispanic Black or African American, had a high school education or less, or had a household income <$50,000 per year than among all workers combined. Fair or poor self-rated health was most common among workers who were Hispanic or were from lower educational attainment and income categories. By occupational group, the prevalence of high levels of economic hardship was highest in farming, fishing, and forestry (18.5%); building and grounds cleaning and maintenance (18.2%); and food preparation and serving (16.0%) and was lowest in the legal occupations (1.2%). Among occupational groups, the prevalence of fair or poor health generally increased with the prevalence of high economic hardship, and almost every occupational group with a high level of economic hardship had a statistically significantly elevated prevalence of fair or poor health compared with that among all workers combined. Given associations between unmet economic needs and health, these findings can be used by policymakers to identify groups of workers with disproportionate economic hardships and develop strategies to enhance economic security and health for all workers.

## Introduction

Economic hardship, the inability to afford basic needs such as food, clothing, and health care, adversely affects health (*1*). A recent study found differences in a subset of economic hardships and health-related social needs by race and ethnicity (*2*). This finding might in part reflect differences in the demographic composition of occupations, given that occupations differ by pay and in benefits such as health insurance and paid time off (*3*,*4*). Given associations between economic hardship and physical (*1*) and mental (*5*) health, the distribution of categories of economic hardship across sociodemographic groups and occupations can help identify where resources are most needed to support the health of workers. This exploratory analysis measured prevalences of economic hardship by sociodemographic and major occupational groups among U.S. adults currently or recently employed for wages or self-employed using 2022–2023 Behavioral Risk Factor Surveillance System (BRFSS) data.

## Methods

### Data Source

BRFSS is conducted annually by all states and three U.S. territories as a random-digit–dialed telephone survey of noninstitutionalized, U.S. civilian residents aged ≥18 years. The BRFSS core survey, administered to all respondents, includes questions about sociodemographic characteristics (including employment), health behaviors, health conditions, and use of health-related services.* BRFSS also offers modules that jurisdictions can opt to administer. The Industry and Occupation (I&O) optional module asks respondents currently employed for wages, self-employed, or out of work for <1 year the question, “What kind of work do you do, for example, registered nurse, janitor, cashier, auto mechanic?”^†^ Participants’ responses are recorded as free text and auto coded to one of 22 two-digit standard occupational classification major groups promulgated by the U.S. Department of Labor.^§^ The 2022 and 2023 Social Determinants of Health/Health Equity (SD/HE) optional modules included questions about economic hardship.^¶^ Thirty-six states and the U.S. Virgin Islands** administered both the I&O and SD/HE modules. Median U.S. BRFSS response rates across jurisdictions in 2022 were 46.3% for landline and 44.7% for cell phone users, and in 2023, median response rates were 54.3% and 40.5%, respectively.^††^ Because BRFSS measures household income rather than individual wages, wage data from the U.S. Department of Labor are reported for occupational groups.^§§^

### Data Analysis

Forms of economic hardship ascertained by the SD/HE module were 1) employment instability, 2) food insecurity, 3) housing insecurity, 4) utility insecurity, 5) lack of reliable transportation, and 6) receipt of food stamps or Supplemental Nutrition Assistance Program (SNAP) benefits.^¶¶^ Two additional economic hardship measures, lack of health insurance and cost as a barrier for needed medical care, were ascertained from the BRFSS core survey section (Table 1). Respondents with at least four types of economic hardship were considered to have high levels of economic hardship.*** Self-reported general health status was also elicited in the core survey.

**TABLE 1:** Economic hardship measures — Behavioral Risk Factor Surveillance System, 36 states and U.S. Virgin Islands, 2022–2023

Hardship	Definition
**Employment instability**	A response of “yes” to the question, “In the past 12 months, have you lost employment or had hours reduced?”
**Food insecurity**	A response of “always,” “usually,” or “sometimes” to the question, “During the past 12 months, how often did the food that you bought not last, and you didn’t have money to get more? Was that…”
**Housing insecurity**	A response of “yes” to the question, “During the last 12 months, was there a time when you were not able to pay your mortgage, rent, or utility bills?”
**Utility insecurity**	A response of “yes” to the question, “During the last 12 months, was there a time when an electric, gas, oil, or water company threatened to shut off services?”
**Lack of reliable transportation**	A response of “yes” to the question, “During the past 12 months, has a lack of reliable transportation kept you from medical appointments, meetings, work, or from getting things needed for daily living?”
**Receiving food stamps or SNAP**	A response of “yes” to the question, “During the past 12 months, have you received food stamps, also called SNAP, the Supplemental Nutrition Assistance Program on an EBT card?”
**Lack of health insurance**	A response of “no coverage of any type” to the question, “What is the current primary source of your health insurance?”
**Cost barrier for needed medical care**	A response of “yes” to the question, “Was there a time in the past 12 months when you needed to see a doctor but could not because you could not afford it?”

**Abbreviations:** EBT = electronic benefits transfer; SNAP = Supplemental Nutrition Assistance Program.

Weighted prevalence and 95% CIs for each type of economic hardship, high levels of economic hardship, and general health were calculated for all workers, for each occupational group, and by sociodemographic category: sex, age group, race and Hispanic or Latino (Hispanic) ethnicity, education level, and household income category. All workers combined comprised a comparison group for sociodemographic categories and occupational groups, hardship, and general health. Prevalence estimates with 95% CIs not overlapping those of the comparison group were considered statistically significantly different. Analyses were conducted with SAS-callable SUDAAN (version 11.0.3; RTI International) to account for the complex survey design. This activity was reviewed by CDC, deemed not research, and was conducted consistent with applicable federal law and CDC policy.^†††^

## Results

According to 2022–2023 BRFSS data, 457,586 respondents were current or recent workers. Among them, 294,606 (64%) were administered the I&O module, 334,241 (73%) were administered the SD/HE module, and 221,681 (48%) were administered both modules. Respondents who reported active military duty status (998; 0.4%) and those for whom occupation was missing, insufficient to code, or with I&O module data indicating that the respondent was not actually working for pay (44,548; 20%) were excluded. In addition, 35,813 participants, including some who were excluded for other reasons, did not respond to any of the six SD/HE questions and were also ineligible, leaving a final study population of 165,060.

Overall, 6.9% of current or recent U.S. workers aged ≥18 years reported at least four types of economic hardship (Table 2). Job loss or reduction in work hours was the most frequently reported economic hardship (16.5%), and lack of reliable transportation the least common (6.7%). The prevalence of several other hardship indicators exceeded 10%, including food insecurity (12.1%), housing insecurity (12.2%), and cost preventing needed medical visits (11.8%).

**TABLE 2:** Prevalence of reported economic hardships among employed* adults, by selected sociodemographic characteristics — Behavioral Risk Factor Surveillance System, 36 states and U.S. Virgin Islands, 2022–2023

Characteristic	No.	Weighted no. (× 1,000)	Prevalence^†^ (95% CI)									
			Lost or reduced employment hours^§^	Food insecurity^¶^	Food stamps or SNAP**	Housing insecurity^††^	Threat to shut off utilities^§§^	Lack of reliable transportation^¶¶^	Could not afford medical care***	No health insurance^†††^	High economic hardship (four or more)	Fair or poor health^§§§^
**All workers combined**	**165,060**	**81,075**	**16.5 (16.1–17.0)**	**12.1 (11.7–12.5)**	**8.2 (7.9–8.6)**	**12.2 (11.9–12.6)**	**7.7 (7.4–8.0)**	**6.7 (6.4–7.0)**	**11.8 (11.5–12.2)**	**9.5 (9.1–9.8)**	**6.9 (6.6–7.2)**	**12.5 (12.1–12.9)**
**Employment status**												
Employed for wages	131,267	64,264	13.3 (12.8–13.7)	11.2 (10.8–11.6)	7.4 (7.0–7.7)	11.1 (10.7–11.5)	7.0 (6.7–7.4)	5.9 (5.6–6.2)	10.8 (10.4–11.2)	7.3 (7.0–7.7)	5.7 (5.4–6.0)	11.9 (11.5–12.3)
Self-employed	28,047	13,241	17.9 (16.7–19.1)^¶¶¶^	11.4 (10.6–12.3)	8.3 (7.5–9.2)	12.6 (11.7–13.5)	8.0 (7.2–8.7)	6.9 (6.2–7.7)	13.4 (12.4–14.4)^¶¶¶^	16.3 (15.2–17.4)^¶¶¶^	7.6 (6.9–8.3)	12.3 (11.3–13.4)
Out of work for <1 year	5,746	3,569	71.6 (69.0–74.0)^¶¶¶^	30.6 (28.1–33.3)^¶¶¶^	23.7 (21.3–26.2)^¶¶¶^	31.3 (28.7–33.9)^¶¶¶^	18.7 (16.6–21.1)^¶¶¶^	20.3 (18.1–22.7)^¶¶¶^	23.8 (21.6–26.1)^¶¶¶^	23.0 (20.7–25.4)^¶¶¶^	26.8 (24.4–29.4)^¶¶¶^	23.8 (21.6–26.2)^¶¶¶^
**Sex**												
Female	78,665	37,606	16.1 (15.4–16.7)	13.8 (13.2–14.4)^¶¶¶^	12.0 (11.4–12.6)^¶¶¶^	14.3 (13.7–14.9)^¶¶¶^	9.0 (8.5–9.5)^¶¶¶^	6.8 (6.4–7.3)	13.0 (12.4–13.6)^¶¶¶^	6.9 (6.5–7.4)	7.9 (7.5–8.4)^¶¶¶^	12.6 (12.0–13.2)
Male	86,395	43,468	16.9 (16.3–17.6)	10.7 (10.2–11.2)	5.0 (4.7–5.4)	10.5 (10.0–10.9)	6.6 (6.2–7.0)	6.5 (6.1–6.9)	10.8 (10.3–11.3)	11.7 (11.2–12.2)^¶¶¶^	6.0 (5.7–6.4)	12.4 (11.9–13.0)
**Age group, yrs**												
18–24	11,388	9,047	27.0 (25.4–28.7)^¶¶¶^	18.6 (17.1–20.1)^¶¶¶^	9.0 (7.8–10.2)	14.4 (13.0–15.9)	6.3 (5.5–7.3)	13.6 (12.4–14.9)^¶¶¶^	16.2 (14.8–17.6)^¶¶¶^	13.3 (12.0–14.7)^¶¶¶^	8.6 (7.6–9.8)^¶¶¶^	13.0 (11.7–14.4)
25–34	25,847	17,544	20.3 (19.2–21.5)^¶¶¶^	15.1 (14.2–16.0)^¶¶¶^	11.7 (10.8–12.7)^¶¶¶^	16.5 (15.6–17.5)^¶¶¶^	9.6 (8.8–10.4)^¶¶¶^	9.3 (8.5–10.1)^¶¶¶^	16.7 (15.8–17.7)^¶¶¶^	13.5 (12.6–14.4)^¶¶¶^	9.7 (9.0–10.5)^¶¶¶^	11.2 (10.5–12.1)
35–49	50,383	25,764	15.3 (14.5–16.0)	12.6 (12.0–13.3)	10.4 (9.8–11.1)^¶¶¶^	14.0 (13.3–14.6)^¶¶¶^	9.7 (9.1–10.3)^¶¶¶^	6.2 (5.8–6.7)	12.3 (11.7–13.0)	10.4 (9.8–11.1)^¶¶¶^	8.1 (7.5–8.6)^¶¶¶^	12.5 (11.9–13.2)
50–64	56,637	22,857	12.4 (11.7–13.2)	8.2 (7.6–8.7)	4.4 (3.9–4.9)	8.2 (7.6–8.8)	5.8 (5.3–6.4)	3.7 (3.3–4.1)	7.8 (7.4–8.3)	6.0 (5.6–6.5)	4.2 (3.8–4.7)	13.1 (12.4–13.8)
≥65	20,805	5,863	10.6 (9.3–11.9)	6.3 (5.2–7.5)	2.0 (1.5–2.5)	4.5 (3.7–5.5)	2.8 (2.3–3.4)	1.8 (1.4–2.4)	3.6 (2.7–4.7)	1.3 (1.0–1.7)	1.4 (1.0–1.9)	13.3 (11.7–15.0)
Race and ethnicity****												
Black or African American	11,567	8,617	21.7 (20.0–23.4)^¶¶¶^	21.4 (19.8–23.1)^¶¶¶^	16.0 (14.7–17.5)	22.9 (21.2–24.6)^¶¶¶^	17.0 (15.5–18.6)^¶¶¶^	10.5 (9.4–11.8)^¶¶¶^	13.1 (11.8–14.5)	7.8 (6.8–8.9)	12.7 (11.4–14.2)^¶¶¶^	14.0 (12.8–15.4)
Hispanic or Latino	17,795	16,329	25.7 (24.4–27.0)^¶¶¶^	21.8 (20.6–23.0)^¶¶¶^	13.1 (12.0–14.1)	19.8 (18.7–20.9)^¶¶¶^	9.3 (8.4–10.2)^¶¶¶^	10.3 (9.4–11.2)^¶¶¶^	18.7 (17.6–19.8)^¶¶¶^	22.6 (21.4–23.9)^¶¶¶^	13.0 (12.0–14.0)^¶¶¶^	19.7 (18.6–20.9)^¶¶¶^
White	125,038	47,105	12.5 (12.1–13.0)	7.4 (7.0–7.7)	5.3 (5.0–5.6)	8.0 (7.6–8.3)	5.8 (5.5–6.1)	4.6 (4.3–4.9)	9.5 (9.1–9.8)	5.9 (5.6–6.2)	4.0 (3.8–4.2)	9.9 (9.6–10.3)
Other race or multiracial	10,660	9,024	16.0 (14.6–17.4)	10.6 (9.5–11.8)	7.3 (6.5–8.2)	10.8 (9.7–11.9)	6.0 (5.2–6.8)	7.3 (6.4–8.3)	10.4 (9.3–11.7)	6.0 (5.2–6.9)	5.6 (4.9–6.5)	11.5 (10.3–12.8)
**Education level**												
Less than high school	7,278	7,471	31.4 (29.3–33.6)^¶¶¶^	33.8 (31.7–36.0)^¶¶¶^	18.0 (16.3–19.9)^¶¶¶^	27.6 (25.7–29.7)^¶¶¶^	14.6 (13.0–16.3)^¶¶¶^	15.8 (14.1–17.6)^¶¶¶^	24.0 (22.1–26.0)^¶¶¶^	32.7 (30.6–34.9)^¶¶¶^	20.3 (18.6–22.1)^¶¶¶^	29.1 (27.1–31.2)^¶¶¶^
High school graduate	35,260	20,203	20.7 (19.7–21.8)^¶¶¶^	17.0 (16.2–17.9) )^¶¶¶^	11.5 (10.7–12.3)^¶¶¶^	15.6 (14.8–16.5)^¶¶¶^	9.1 (8.4–9.8)^¶¶¶^	8.7 (8.1–9.3)^¶¶¶^	14.7 (13.9–15.6)^¶¶¶^	13.0 (12.2–13.8) )^¶¶¶^	9.2 (8.5–9.9)^¶¶¶^	14.2 (13.5–15.0)^¶¶¶^
Some college or technical school	42,375	24,008	17.8 (16.9–18.7)	11.2 (10.6–11.8)	9.1 (8.5–9.7)	13.4 (12.7–14.1)^¶¶¶^	9.2 (8.6–9.9)^¶¶¶^	6.9 (6.4–7.4)	12.2 (11.6–12.9)	7.7 (7.1–8.3)	7.1 (6.6–7.7)	12.5 (11.8–13.2)
College graduate or more	79,833	29,224	8.7 (8.3–9.2)	3.9 (3.6–4.2)	2.8 (2.6–3.1)	5.0 (4.7–5.4)	3.8 (3.5–4.1)	2.7 (2.5–3.0)	6.3 (6.0–6.7)	2.8 (2.6–3.1)	1.7 (1.5–1.9)	7.1 (6.6–7.6)
**Annual household income**												
<$25,000	10,240	6,418	41.8 (39.8–43.8)^¶¶¶^	43.3 (41.3–45.3)^¶¶¶^	31.8 (29.9–33.8)^¶¶¶^	38.3 (36.3–40.3)^¶¶¶^	21.0 (19.4–22.8)^¶¶¶^	21.2 (19.6–22.9)^¶¶¶^	29.3 (27.4–31.2)^¶¶¶^	29.4 (27.5–31.4)^¶¶¶^	30.7 (28.8–32.6)^¶¶¶^	27.8 (26.0–29.7)^¶¶¶^
$25,000–$34,999	12,222	6,929	31.2 (29.3–33.2)^¶¶¶^	29.0 (27.2–30.9)^¶¶¶^	21.9 (20.2–23.7)^¶¶¶^	28.0 (26.3–29.7)^¶¶¶^	16.9 (15.4–18.5)^¶¶¶^	14.5 (13.2–16.0)^¶¶¶^	23.6 (21.9–25.4)^¶¶¶^	21.0 (19.3–22.8)^¶¶¶^	17.7 (16.3–19.3)^¶¶¶^	20.4 (18.8–22.0)^¶¶¶^
$35,000–$49,999	16,991	8,170	21.8 (20.3–23.4)^¶¶¶^	19.0 (17.7–20.4)^¶¶¶^	12.3 (11.1–13.6)^¶¶¶^	19.0 (17.7–20.4)^¶¶¶^	12.4 (11.3–13.6)^¶¶¶^	8.9 (7.9–9.9)^¶¶¶^	17.4 (16.2–18.7)^¶¶¶^	13.3 (12.1–14.6)^¶¶¶^	10.0 (9.0–11.2)^¶¶¶^	16.4 (15.2–17.7)^¶¶¶^
$50,000–$74,999	24,479	11,003	15.0 (13.8–16.3)	9.7 (8.8–10.7)	5.6 (4.7–6.6)	12.6 (11.4–13.8)	8.7 (7.7–9.8)	5.6 (4.8–6.4)	12.6 (11.6–13.6)	8.0 (7.2–8.9)	4.7 (3.9–5.6)	12.2 (11.2–13.2)
$75,000–$99,999	23,051	10,232	10.1 (9.3–11.1)	4.7 (4.1–5.3)	2.5 (2.0–3.0)	5.9 (5.3–6.6)	4.6 (4.0–5.2)	3.4 (2.9–4.0)	8.6 (7.8–9.5)	5.0 (4.3–5.9)	1.7 (1.4–2.1)	9.3 (8.4–10.3)
$100,000–$199,999	42,705	19,278	7.6 (7.0–8.3)	2.0 (1.7–2.3)	1.4 (1.1–1.7)	3.1 (2.7–3.5)	2.9 (2.5–3.3)	1.8 (1.5–2.1)	4.5 (4.1–5.1)	2.6 (2.3–2.9)	0.7 (0.5–1.0)	7.0 (6.4–7.7)
≥$200,000	15,690	7,836	5.1 (4.4–6.0)	0.8 (0.6–1.1)	0.8 (0.4–1.3)	1.2 (0.8–1.7)	1.5 (0.9–2.3)	0.8 (0.6–1.1)	2.1 (1.6–2.7)	1.4 (1.1–1.8)	NR^††††^	4.3 (3.7–5.0)

**Abbreviations:** EBT = electronic benefits transfer; NR = not reportable; SNAP = Supplemental Nutrition Assistance Program.

* Respondents who reported being “employed for wages,” “self-employed,” or “out of work for less than 1 year” were included in the analyses.

^†^ Unadjusted, weighted prevalence estimates.

^§^ Respondents who answered “yes” to the question, “In the past 12 months, have you lost employment or had hours reduced?”

^¶^ Respondents who answered “always,” “usually,” or “sometimes” to the question, “During the past 12 months, how often did the food that you bought not last, and you didn’t have money to get more? Was that…”

** Respondents who answered “yes” to the question, “During the past 12 months, have you received food stamps, also called SNAP, the Supplemental Nutrition Assistance Program on an EBT card?”

^††^ Respondents who answered “yes” to the question, “During the last 12 months, was there a time when you were not able to pay your mortgage, rent, or utility bills?”

^§§^ Respondents who answered “yes” to the question, “During the last 12 months, was there a time when an electric, gas, oil, or water company threatened to shut off services?”

^¶¶^ Respondents who answered “yes” to the question, “During the past 12 months, has a lack of reliable transportation kept you from medical appointments, meetings, work, or from getting things needed for daily living?”

*** Respondents who answered “yes” to the question, “Was there a time in the past 12 months when you needed to see a doctor but could not because you could not afford it?”

^†††^ Respondents who answered “no coverage of any type” to the question, “What is the current primary source of your health insurance?”

^§§§^ Respondents who answered “fair” or “poor” to the question, “Would you say that in general your health is excellent, very good, good, fair, or poor?”

^¶¶¶^ Statistically significant elevated prevalence of hardship in the occupational group compared with all workers.

**** Persons of Hispanic or Latino (Hispanic) origin might be of any race but are categorized as Hispanic; all racial groups are non-Hispanic.

^††††^ Relative SE >30%.

### Differences by Sociodemographic Characteristics

Compared with all workers, for each type of economic hardship, prevalence was statistically significantly elevated among respondents who were out of work for <1 year, were aged 18–49 years (particularly 18–24 years), were Hispanic or non-Hispanic Black or African American (Black), had a high school education or less, or had household incomes <$50,000. Prevalences were statistically significantly higher among female than male workers for five of the eight categories of hardship: food insecurity (13.8% of women versus 10.7% of men), receipt of food stamps or SNAP (12.0% versus 5.0%), housing insecurity (14.3% versus 10.5%), threatened utility cutoff (9.0% versus 6.6%), cost preventing needed medical care (13.0% versus 10.8%), and high levels of economic hardship (7.9% versus 6.0%). However, a higher percentage of male workers lacked health insurance (11.7%) than did female workers (6.9%). Compared with all workers combined, fair or poor general health was statistically significantly more common among respondents who were out of work for <1 year, were Hispanic, had a high school education or less, or had household incomes <$50,000.

### Differences by Occupational Group

High levels of economic hardship were most common in the farming, fishing, and forestry (18.5%) occupational group; building and grounds cleaning and maintenance (18.2%); and food preparation and serving (16.0%) and were lowest for the legal occupations (1.2%) (Table 3). Prevalences of high levels of economic hardship exceeded 10% for three additional occupational groups: health care support (14.1%), construction and extraction (11.6%), and transportation and materials manufacturing (10.6%). U.S. Department of Labor data indicate 2023 mean annual wages were <$50,000 and median annual wages were <$45,000 for all occupational groups with high levels of economic hardship prevalences (≥10%), except construction and extraction (mean annual wage = $61,500; median = $55,680). Prevalences of the two health care measures, lacking health insurance and cost preventing needed medical care, were statistically significantly elevated for each occupational group with a high level of economic hardship except health care support. The prevalence of fair or poor health generally increased with the percentage of the occupation experiencing high economic hardship. With the exception of construction and extraction, this prevalence was statistically significantly elevated in every occupational group with a high level of economic hardship, compared with all workers combined.

**TABLE 3:** Prevalence of economic hardships among employed* adults, by occupation and selected sociodemographic characteristics — Behavioral Risk Factor Surveillance System, 36 states and U.S. Virgin Islands, 2022–2023

Occupational group annual wage (mean; median)^§^	No.	Weighted no. (× 1,000)	Prevalence^†^ (95% CI)									
			High level of economic hardship (four or more)	Lost or reduced employment hours^¶^	Food insecurity**	Food stamps or SNAP^††^	Housing insecurity^§§^	Notice of potential utility shut off^¶¶^	Lack of reliable transportation***	Could not afford medical care^†††^	No health insurance^§§§^	Fair or poor health^¶¶¶^
**All workers combined ($65,470; $48,060)**	**165,060**	**81,075**	**6.9 (6.6–7.2)**	**16.5 (16.1–17.0)**	**12.1 (11.7–12.5)**	**8.2 (7.9–8.6)**	**12.2 (11.9–12.6)**	**7.7 (7.4–8.0)**	**6.7 (6.4–7.0)**	**11.8 (11.5–12.2)**	**9.5 (9.1–9.8)**	**12.5 (12.1–12.9)**
Farming, fishing, and forestry ($39,970; $35,520)	1,249	619	18.5 (12.5–25.9)****	31.8 (25.1–39.1)****	32.1 (25.2–39.6)****	20.2 (13.4–28.4)****	24.5 (18.1–31.9)****	11.7 (6.3–19.3)	13.7 (8.1–21.1)****	21.2 (15.1–28.4)****	28.8 (23.1–35.0)****	30.1 (23.7–37.2)****
Building and grounds cleaning and maintenance ($38,320; $35,990)	5,635	3,447	18.2 (16.1–20.3)****	25.7 (23.4–28.2)****	30.6 (28.1–33.2)****	18.1 (15.9–20.5)****	26.3 (23.9–28.7)****	12.1 (10.2–14.3)****	12.3 (10.6–14.1)****	23.2 (20.9–25.7)****	27.2 (24.6–29.9)****	23.5 (21.1–26.0)****
Food preparation and serving ($34,490; $32,240)	4,989	3,065	16.0 (13.9–18.2)****	31.6 (28.7–34.6)****	24.6 (22.2–27.2)****	17.1 (14.9–19.4)****	22.2 (19.9–24.7)****	12.9 (11.1–15.0)****	16.2 (14.0–18.6)****	21.4 (19.1–23.9)****	22.8 (20.1–25.6)****	20.9 (18.5–23.4)****
Health care support ($38,220; $36,140)	4,956	2,902	14.1 (11.7–16.8)****	23.8 (21.0–26.7)****	25.5 (22.7–28.5)****	23.1 (20.4–26.0)****	25.0 (22.2–28.0)****	14.8 (12.6–17.2)****	12.8 (10.6–15.4)****	18.5 (15.9–21.4)****	9.5 (7.7–11.5)	19.1 (16.3–22.2)****
Construction and extraction ($61,500; $55,680)	10,239	5,789	11.6 (10.3–13.1)****	25.5 (23.5–27.5)****	16.5 (14.9–18.2)****	6.6 (5.6–7.7)	16.9 (15.3–18.5)****	9.7 (8.4–11.0)****	9.8 (8.5–11.3)****	17.3 (15.6–19.0)****	24.7 (22.7–26.8)****	13.3 (11.9–14.9)
Transportation and material moving ($46,690; $40,050)	9,315	5,311	10.6 (9.2–12.2)****	25.3 (23.2–27.4)****	18.9 (17.1–20.8)****	11.9 (10.5–13.4)	19.1 (17.3–21.0)****	10.6 (9.2–12.2)****	9.5 (8.1–11.1)****	15.1 (13.5–16.8)****	13.7 (12.2–15.3)****	17.4 (15.8–19.1)****
Personal care and service ($38,430; $34,260)	3,111	1,723	8.8 (6.9–11.0)	20.6 (17.3–24.2)****	18.5 (15.2–22.2)****	14.4 (11.9–17.1)	17.8 (14.5–21.4)****	9.6 (7.7–11.9)	7.4 (5.7–9.5)	14.1 (11.3–17.4)	10.2 (7.9–12.8)	14.0 (11.1–17.2)
Sales and related ($53,280; $36,760)	13,881	7,508	8.3 (7.1–9.6)	21.0 (19.3–22.8)****	13.3 (12.1–14.6)	10.9 (9.5–12.4)	13.3 (11.9–14.8)	8.6 (7.4–9.8)	7.8 (6.9–8.8)	13.1 (11.8–14.5)	9.2 (8.0–10.4)	13.0 (11.7–14.3)
Production ($47,620; $43,630)	6,685	3,267	8.2 (6.7–10.0)	20.8 (18.5–23.1)****	15.9 (14.0–17.9)****	8.3 (6.8–10.0)	15.0 (13.0–17.1)****	9.7 (8.0–11.6)****	9.0 (7.2–11.0)****	13.0 (11.4–14.8)	11.9 (10.2–13.8)****	17.4 (15.5–19.4)****
Office and administrative support ($47,940; $44,480)	14,019	7,129	6.7 (5.7–7.7)	16.5 (15.0–18.2)	12.8 (11.5–14.2)	10.4 (9.1–11.8)****	14.2 (12.9–15.6)****	9.1 (8.0–10.2)****	6.2 (5.3–7.1)	12.9 (11.6–14.3)	6.3 (5.3–7.5)	12.9 (11.6–14.2)
Installation, maintenance, and repair ($58,500; $53,920)	5,474	3,261	6.2 (5.0–7.7)	16.0 (14.0–18.2)	11.4 (9.7–13.4)	4.8 (3.7–6.1)	12.1 (10.4–13.9)	8.0 (6.5–9.6)	5.9 (4.7–7.3)	11.4 (9.6–13.3)	13.8 (11.7–16.2)****	12.9 (11.1–14.9)
Protective service ($57,710; $47,760)	3,057	1,750	4.3 (2.9–6.2)	14.9 (10.3–20.6)	10.6 (8.3–13.3)	7.3 (4.9–10.3)	10.6 (7.8–13.9)	6.0 (4.4–7.9)	4.6 (3.3–6.2)	6.8 (5.1–8.8)	3.9 (2.7–5.5)	9.8 (7.9–12.1)
Community and social services ($58,980; $52,000)	4,381	1,556	3.4 (2.4–4.7)	9.8 (8.2–11.6)	8.5 (6.4–11.0)	8.0 (5.8–10.6)	9.1 (7.3–11.1)	7.3 (5.5–9.5)	5.1 (3.6–7.0)	8.7 (7.3–10.4)	2.5 (1.7–3.4)	11.0 (9.3–12.8)
Arts, design, entertainment, sports, and media ($75,520; $51,660)	3,882	1,922	3.2 (2.2–4.4)	18.5 (15.4–21.9)	7.8 (5.2–11.1)	4.6 (2.7–7.4)	6.9 (5.3–8.7)	4.3 (3.2–5.7)	5.6 (4.2–7.2)	11.7 (9.0–15.0)	6.0 (4.7–7.6)	10.3 (7.7–13.5)
Business and financial operations ($90,580; $79,050)	10,063	4,723	3.1 (2.2–4.1)	10.0 (8.6–11.6)	4.9 (4.0–6.1)	3.3 (2.6–4.0)	6.6 (5.5–7.9)	5.3 (4.1–6.6)	3.0 (2.3–3.8)	8.3 (7.2–9.6)	3.5 (2.6–4.6)	7.6 (6.6–8.7)
Management ($137,750; $116,880)	19,965	8,114	2.9 (2.4–3.5)	7.9 (7.1–8.7)	5.0 (4.3–5.6)	3.4 (2.8–3.9)	5.9 (5.2–6.7)	4.8 (4.0–5.7)	3.5 (2.8–4.3)	7.1 (6.4–7.9)	5.3 (4.5–6.2)	8.7 (7.7–9.7)
Education, training, and library ($66,400; $59,940)	11,544	4,259	2.9 (2.3–3.7)	8.6 (7.3–9.9)	6.2 (5.3–7.3)	6.8 (5.6–8.2)	7.8 (6.6–9.2)	5.5 (4.5–6.7)	4.0 (3.2–5.0)	7.1 (6.1–8.1)	2.5 (1.9–3.3)	8.9 (7.6–10.4)
Health care practitioners and technical ($102,060; $80,820)	13,770	6,110	2.8 (2.2–3.5)	9.6 (8.5–10.7)	5.2 (4.5–6.0)	4.3 (3.5–5.3)	6.4 (5.5–7.3)	5.0 (4.2–5.8)	2.8 (2.3–3.5)	7.9 (6.9–9.0)	3.7 (2.9–4.6)	8.1 (6.8–9.7)
Computer and mathematical ($113,140; $104,200)	7,282	3,703	1.7 (1.2–2.3)	9.3 (7.5–11.3)	3.3 (2.6–4.1)	1.6 (1.1–2.3)	3.8 (3.0–4.7)	3.9 (3.0–5.0)	3.7 (2.7–4.8)	7.1 (5.8–8.6)	2.8 (2.0–3.8)	8.9 (7.6–10.5)
Architecture and engineering ($99,090; $91,420)	5,624	2,561	1.3 (0.7–2.0)	6.8 (5.5–8.3)	4.0 (2.6–5.8)	1.3 (0.8–2.1)	2.7 (2.0–3.5)	2.4 (1.7–3.4)	2.2 (1.6–3.1)	4.2 (3.3–5.4)	1.6 (1.0–2.5)	7.3 (5.9–9.0)
Legal ($133,820; $99,220)	2,553	1,071	1.2 (0.7–2.0)	6.3 (4.3–8.8)	2.0 (1.1–3.3)	NR^††††^	3.6 (2.6–4.8)	2.9 (2.0–4.0)	2.2 (1.4–3.3)	3.8 (2.7–5.3)	1.8 (1.0–3.0)	6.1 (4.5–7.9)
Life, physical, and social science ($87,870; $52,000)	3,386	1,286	NR^††††^	9.8 (7.4–12.7)	5.2 (3.1–8.1)	NR^††††^	4.6 (3.2–6.5)	5.3 (3.1–8.3)	4.6 (2.7–7.2)	5.5 (4.3–7.1)	1.6 (1.0–2.5)	5.8 (4.4–7.4)

**Abbreviations:** EBT = electronic benefits transfer; NR = not reportable; SNAP = Supplemental Nutrition Assistance Program.

* Respondents who reported being “employed for wages,” “self-employed,” or “out of work for less than 1 year” were included in the analyses.

^†^ Unadjusted, weighted prevalence estimates.

^§^ May 2023 National Occupational Employment and Wage Estimates: U.S. Bureau of Labor Statistics

^¶^ Respondents who answered “yes” to the question, “In the past 12 months, have you lost employment or had hours reduced?”

** Respondents who answered “always,” “usually,” or “sometimes” to the question, “During the past 12 months, how often did the food that you bought no last, and you didn’t have money to get more? Was that…”

^††^ Respondents who answered “yes” to the question, “During the past 12 months, have you received food stamps, also called SNAP, the Supplemental Nutrition Assistance Program on an EBT card?”

^§§^ Respondents who answered “yes” to the question, “During the last 12 months, was there a time when you were not able to pay your mortgage, rent, or utility bills?”

^¶¶^ Respondents who answered “yes” to the question, “During the last 12 months, was there a time when an electric, gas, oil, or water company threatened to shut off services?”

*** Respondents who answered “yes” to the question, “During the past 12 months, has a lack of reliable transportation kept you from medical appointments, meetings, work, or from getting things needed for daily living?”

^†††^ Respondents who answered “yes” to the question, “Was there a time in the past 12 months when you needed to see a doctor but could not because you could not afford it?”

^§§§^ Respondents who answered “no coverage of any type” to the question, “What is the current primary source of your health insurance?

^¶¶¶^ Respondents who answered “fair” or “poor” to the question, “Would you say that in general your health is excellent, very good, good, fair, or poor?”

**** Statistically significant elevated prevalence of hardship in the occupational group compared with all workers.

^††††^ Relative SE >30%.

### Sociodemographic Characteristics of Occupational Groups with High Levels of Economic Hardship

Several occupational groups with the highest prevalences of high levels of economic hardship had disproportionate percentages of workers from demographic groups with high prevalences of economic hardship and fair or poor health (Supplementary Table). Within the farming, fishing, and forestry occupational group, 66.5% of workers reported Hispanic ethnicity (compared with 20.6% of all workers), and 54.8% had not completed high school (compared with 9.5% of all workers). Within the building and grounds cleaning and maintenance occupational group, 52.7% of workers reported Hispanic ethnicity, and 37.4% had not completed high school. Prevalences of each economic hardship indicator, high economic cost, and fair or poor health were statistically significantly elevated among both Hispanic workers and those who had not completed high school. Each economic hardship measure was statistically significantly elevated in young workers, and a majority of economic hardship measures were statistically significantly elevated among female and Black workers; however, the prevalence of fair or poor health was not significantly elevated in these demographic groups. The highest prevalence of workers aged <35 years (57.6% versus 32.6% of all workers) was in the food preparation and serving industry. The health care support occupational group had the highest prevalence of female workers (83.0% versus 46.6% of all workers) and the second highest prevalence of non-Hispanic Black workers (20.1% versus 10.5% of all workers).

## Discussion

This exploratory study found substantial differences in the prevalence of high economic hardship among sociodemographic groups and by major occupational group. Generally, low-wage jobs are less likely to offer employment benefits such as health insurance (*6*) and paid sick leave (*7*). However, wage gaps are not always concordant with differences in levels of benefits provided (*5*). In this analysis, the mean income of construction and extraction workers was only slightly below the all-occupation mean, but prevalences of every economic hardship except receiving food stamps or SNAP were significantly higher. Moreover, the relationship between income and specific economic hardships is complex (*5*). Poor employment quality (including insufficient income and material benefits, employment instability, and occupational exposures) has been associated with poor health (self-rated, mental, and occupational injury) (*8*), and in these analyses, workers in occupational groups with high economic hardships were more likely to report fair or poor self-rated health. In addition, these occupational groups often included high proportions of workers from demographic groups with high economic hardship and suboptimal health. Increased health insurance coverage among low-income adults is associated with better management of chronic health conditions (*8*). Further, the observation in this study that more workers in occupational groups with lower mean incomes could not afford needed medical care suggests that adequate wages might play a role in keeping workers healthy. Addressing economic hardships, especially high health care costs and lack of insurance, is important for enhancing economic security and health for all workers.

### Limitations

The findings in this report are subject to at least nine limitations. First, BRFSS data are cross-sectional, with timing and duration of economic hardships unknown, limiting causal inference. Second, 2022 BRFSS data might reflect early COVID-19 pandemic employment disruptions that affected sociodemographic and occupational groups differently. Third, several economic hardship indicators overlap, leading to some redundancy in the high levels of economic hardship metric. Fourth, BRFSS data are self-reported and subject to recall and social desirability biases. Fifth, missing or misclassified survey data could bias results. Sixth, major occupational groups are broad, with components differing by sociodemographic composition, wages, and benefits. Seventh, the respondent’s occupation might not be the source of health insurance or the sole source of household income. Eighth, results were from 36 states and the U.S. Virgin Islands and were not nationally representative. Finally, this exploratory analysis of BRFSS data had no prior guiding hypotheses.

### Implications for Public Health Practice

The findings that some sociodemographic and occupational groups disproportionately bear the cost of economic hardship, including high costs preventing health care, lack of health insurance, and fair or poor health, highlight gaps that affect worker health and the sustainability of employment. The public health community, social service and health care systems, and policymakers can use this information to create tailored programs to reduce economic hardships that lead to differential adverse health outcomes. For example, increased health insurance coverage among adults with lower incomes is associated with better management of chronic health conditions (*8*). Further, the observation in this study that more workers in occupational groups with lower mean incomes could not afford needed medical care suggests that adequate wages might play a role in keeping workers healthy. Data from future surveys can be used to monitor trends in economic hardship among workers and evaluate intervention efficacy.

## Footnotes

*
CDC - About BRFSS

†
Statistical Brief for the Behavioral Risk Factor Surveillance System (BRFSS) Industry and Occupation Optional Module 2022

§
2010 Standard Occupational Classification System: U.S. Bureau of Labor Statistics

¶ 2022 BRFSS Questionnaire and 2023 BRFSS Questionnaire

** The U.S. Virgin Islands and the following states administered both the I&O and the SD/HE modules in 2022, 2023, or both years: Arizona, California, Connecticut, Delaware, Florida, Georgia, Idaho, Illinois, Indiana, Iowa, Kansas, Kentucky, Louisiana, Maryland, Massachusetts, Michigan, Minnesota, Mississippi, Missouri, Montana, Nebraska, New Hampshire, New Jersey, New Mexico, New York, North Dakota, Oklahoma, Rhode Island, Tennessee, Texas, Utah, Vermont, Washington, West Virginia, Wisconsin, and Wyoming.

†† Response rates overall and by jurisdiction: CDC - 2022 BRFSS Survey Data and Documentation and CDC - 2023 BRFSS Survey Data and Documentation.

§§
May 2023 National Occupational Employment and Wage Estimates: U.S. Bureau of Labor Statistics

¶¶ High economic hardship was defined as reporting at least four specific economic hardships. This cutoff acknowledges overlaps in content of some economic hardship metrics included in BRFSS (food insecurity and receipt of food stamps or SNAP, and housing insecurity and utility insecurity). A cutoff of four hardships identifies respondents with hardships in a minimum of two different domains.

*** Receiving food stamps or SNAP has also been considered a health-related social need by researchers.

††† 45 C.F.R. part 46.102(l)(2), 21 C.F.R. part 56; 42 U.S.C. Sect. 241(d); 5 U.S.C. Sect. 552a; 44 U.S.C. Sect. 3501 et seq.

## References

[1] Friedline T, Chen Z, Morrow S. Families’ financial stress & well-being: the importance of the economy and economic environments. J Fam Econ Issues 2021;42(Suppl 1):34–51. 10.1007/s10834-020-09694-9 32837140 PMC7362317

[2] Town M, Eke P, Zhao G, Racial and ethnic differences in social determinants of health and health-related social needs among adults—Behavioral Risk Factor Surveillance System, United States, 2022. MMWR Morb Mortal Wkly Rep 2024;73:204–8. 10.15585/mmwr.mm7309a3 38451870 PMC10932584

[3] Gradín C. Segregation of women into low-paying occupations in the United States. Appl Econ 2020;52:1905–20. 10.1080/00036846.2019.1682113

[4] Kristal T, Cohen Y, Navot E. Benefit inequality among American workers by gender, race and ethnicity, 1982–2015. Sociol Sci 2018;5:461–88. 10.15195/v5.a20

[5] Heflin CM, Iceland J. Poverty, material hardship and depression. Soc Sci Q 2009;90:1051–71. 10.1111/j.1540-6237.2009.00645.x 25530634 PMC4269256

[6] National Library of Medicine, National Institutes of Health. Access to healthcare and disparities in access. Rockville, MD: National Institutes of Health, National Library of Medicine; 2021. https://www.ncbi.nlm.nih.gov/books/NBK578537/

[7] Bureau of Labor Statistics, US Department of Labor. TED: the economics daily. Higher paid workers more likely than lower paid workers to have paid leave benefits in 2022. Washington, DC: US Department of Labor, Bureau of Labor Statistics; 2023. https://www.bls.gov/opub/ted/2023/higher-paid-workers-more-likely-than-lower-paid-workers-to-have-paid-leave-benefits-in-2022.htm

[8] Peckham T, Fujishiro K, Hajat A, Flaherty BP, Seixas N. Evaluating employment quality as a determinant of health in a changing labor market. RSF 2019;5:258–81. 10.7758/rsf.2019.5.4.09 31548990 PMC6756794

